# Transmitted Drug Resistance Mutations in Antiretroviral-Naïve Injection Drug Users with Chronic HIV-1 Infection in Iran

**DOI:** 10.1371/journal.pone.0126955

**Published:** 2015-05-11

**Authors:** Arash Memarnejadian, Shahoo Menbari, Seyed Ali Mansouri, Leila Sadeghi, Rouhollah Vahabpour, Mohammad Reza Aghasadeghi, Ehsan Mostafavi, Mohammad Abdi

**Affiliations:** 1 Department of Hepatitis and AIDS, Pasteur Institute of Iran, Tehran, Iran; 2 Department of Pathology and Medical Laboratory Sciences, Faculty of Paramedicine, Kurdistan University of Medical Sciences, Sanandaj, Iran; 3 Department of Microbiology, Faculty of Sciences, Islamic Azad University, Qom, Iran; 4 Department of Microbiology, Faculty of Sciences, Islamic Azad University, Karaj, Iran; 5 Department of Virology, Faculty of Public Health, Tehran University of Medical Sciences, Tehran, Iran; 6 Department of Epidemiology, Pasteur Institute of Iran, Tehran, Iran; Centro de Biología Molecular Severo Ochoa (CSIC-UAM), SPAIN

## Abstract

The growing incidence and transmission of drug resistant HIV-1 strains due to widespread use of antiretroviral therapy (ART) can jeopardize the success of first-line ART. While there is a known moderate prevalence of transmitted drug resistance (TDR) among newly infected Iranians, no data exist about the rate of these primary resistance mutations among the ART-naïve, chronically infected individuals who are, in fact, the main candidates for ART initiation. To address this issue, we collected blood samples from 40 ART-naïve injection drug-users (IDUs) with chronic HIV-1 infection (seroconversion time ranging from 2 to 9 years) living in Sanandaj, Iran, followed by sequencing of the protease and reverse-transcriptase regions from their HIV-1 genome. Phylogenetic analyses of the sequenced regions revealed that all samples were CRF35_AD. Transmitted resistance mutations were interpreted as surveillance drug-resistant mutations (SDRMs) based on the world health organization (WHO) algorithm. The frequency of SDRMs to any class of antiretroviral drugs was 15%, which included mutations to nucleoside reverse transcriptase inhibitors (NRTIs, 10%), with M41L and M184V as the most common (5%), and non-nucleoside reverse transcriptase inhibitors (NNRTIs, 5%), with K103N as the only detected mutation (5%). Although not in the WHO SDRMs list, several minor protease inhibitor resistant mutations listed in the International Antiviral Society-USA panel were identified, of which M36I, H69K, L89M/V/I (each one 100%) and K20R/T (92.5%) can be considered as polymorphic signatures for CRF35_AD.The relatively high rate of TDR mutations in our study raises concerns about the risk of treatment failure in chronically infected IDUs of Sanandaj city. These results suggest that routine resistance testing should be considered before the therapy initiation in this area. Additional surveillance studies are required to generalize this deduction to other cities of Iran.

## Introduction

Incorporation of highly active antiretroviral therapy (HAART) into clinical practice has resulted in a 60% to 80% decline in rates of Acquired Immunodeficiency Syndrome (AIDS) death and hospitalization [1]. However, an adverse consequence of antiretroviral therapy (ART) is the emergence and selection of antiretroviral resistant mutant variants, the major cause of ART failure in the treatment of AIDS[2–3]. These variants have become widespread, in drug-treated and untreated individuals infected with human immunodeficiency virus (HIV), and have compromised the therapeutic options in drug-naïve infected persons [4]. Transmission of resistant mutants from drug-experienced patients to newly infected drug-naïve individuals was initially noted in developed countries with good access to antiretroviral drugs [5–8]. More recently, ART scaling-up in resource-limited settings is resulting in the occurrence of primary mutations in developing countries, as well [2, 9]. The world health organization (WHO) recommends periodic surveillance of transmitted drug resistance (TDR) mutations in drug-naïve, recently infected individuals in distinct geographical areas[10]. Furthermore, current treatment guidelines recommend routine laboratory testing to assess drug resistance-associated mutations (DRAMs) in patients with acute and chronic infections prior to ART initiation to optimize the treatment regimen [11–13]. These are particularly recommended in countries scaling up ART and in areas where primary resistance has been consistently documented[12, 14]. Lack of testing for baseline resistance, in addition to, other factors including interruption in treatment due to disruption in drug supply, or as a result of financial restrictions and improper administration of drug regimens are the major causes for the occurrence and expansion of drug resistance in developing countries [2].

Since 1986, when the first HIV-1 positive case was reported in Iran, the number of people living with HIV/AIDS has been increased to 27041 cases that have been registered by the end of 2013. In 2015, the number of Iranians with HIV/AIDS is estimated to be more than 120,000[15]. Although an increasing incidence of sexual transmission of HIV has been recently observed, the main route of HIV transmission in Iran is still through injection, so that injection drug users (IDUs) comprise the majority of the HIV-infected population[15–16]. ART in Iran was initiated in2004; however, drug resistance testing is not a prerequisite for ART initiation yet, likely due to limited financial resources and lack of accumulated data on primary resistance. Two studies that used the WHO surveillance drug-resistant mutation(SDRM) list [17] found TDR frequencies of 4.3% [18] and 5.1% [19] among 47 and 39 newly infected ART-naïve Iranian subjects, respectively, with one noting a moderate predicted prevalence (5–15%) throughout the country [18]. Another study using the Stanford drug resistance database[20]noted a TDR mutation frequency of 6.7% among30 ART-naïve subjects living in Tehran city, irrespective of the length of infection [21]. Apart from these, we are aware of no other study addressing the existence of primary drug resistance in Iran and, more importantly, no data is available regarding the prevalence of baseline resistance in ART-naïve Iranians with long-term established (chronic) HIV-1 infection. This lack of information is highlighted in relation to the fact that people with chronic HIV-1 infection are typically candidates for initiation of ART. The objective of this study, therefore, is to determine the frequency of TDR mutations in a chronically infected ART-naïve population in Iran.

## Methods

### Study subjectsand sample collection

For this cross-sectional study, we enrolled forty HIV-1 positive IDUs who visited Voluntary Counseling and Testing (VCT) centers of Sanandaj, a western city of Iran, in 2011. All participants had been diagnosed as HIV-1 positive before 2009 (seroconversion time ranging from 2 to 9 years), were antiretroviral-naïve and based upon the attending physician’s decision became candidates to initiate HAART at the time of enrollment. Blood (5 mL) was collected from the participants and prepared plasma samples were stored at -70°C. Participants were provided with a detailed questionnaire including socio-demographic questions and blood samples for laboratory results obtained on the same day.

### Ethics statement

Study procedure was approved by institutional Ethics Committee of the Pasteur Institute of Iran. All 40 participants signed a written informed consent before blood sample collection.

### Determination of drug resistance mutations and HIV-1 subtype

The drug resistance genotypic testing protocol previously described [22]was used with some modifications. Briefly, viral RNA was extracted from 140 μl of plasma by QIA amp viral RNA mini kit (Qiagen, Düsseldorf, Germany), according to the manufacturer’s protocol. WholeHIV-1 protease fragment (PR, nucleotide positions of 2285 to 2550 in HIV-1 HXB2 sequence) and N-terminal fragment of reverse transcriptase (RT, nucleotide positions of 2673 to 3269 in HIV-1 HXB2 sequence)were amplified using specific primers[22]and SuperScript III one-step RT-PCR kit with platinum Taq (Life Technologies, Carlsbad, CA), and further re-amplified in nested PCR using i-Taq Maxime PCR Premix (Intron Biotechnologies, Kyungki-Do, Korea). Final PCR products were purified by QIA quick PCR purification kit (Qiagen, Düsseldorf, Germany) and sequenced using BigDye terminator cycle sequencing kit v3.1and ABI PRISM 3130 Genetic Analyzer (Applied Biosystems, Foster City, CA). Obtained sequences of each sample were assembled to HXB2 PR-RT (GenBank accession number: K03455) as the reference sequence by CLC Main Workbench 5.5 software (CLC bio, Boston, MA). TDR mutations were determined based on the WHO-recommended surveillance drug-resistant mutations (SDRMs) list [17]. To determine the subtypes, PR-RT sequences (1017 bp) were aligned against the subtype and CRF reference sequences retrieved from the Los Alamos HIV-1 database (http://www.hiv.lanl.gov/) by Clustal W. A phylogenetic tree was constructed using the neighbor-joining method with 1000 bootstraps, as implemented in MEGA 5 software [23]. To validate the results, we further submitted PR-RT sequences to the REGA HIV-1 Subtyping Tool V3 [24]. PR-RT sequences obtained in this study (1017 bp) were deposited in the GenBank database and are available under the accession numbers [GenBank: KF544024-KF544063].

## Results

**Fig 1:**
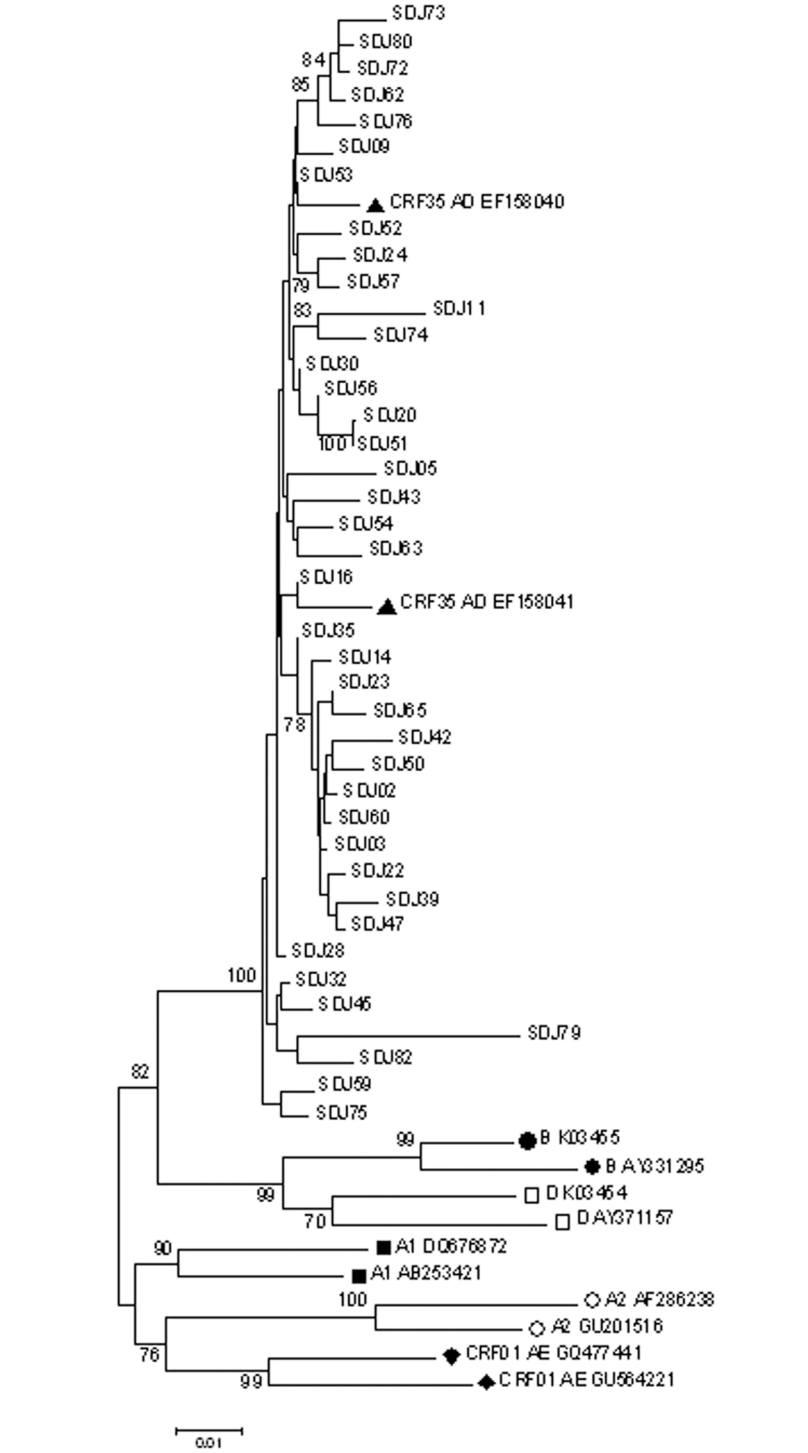
Summarized phylogenetic tree of 40 Sanandaj (SDJ) samples based on protease and reverse-transcriptase (PR-RT, 1017 bp) sequences. Tree was constructed using neighbor-joining method with 1000 replicates. As a summarized representation, only reference sequences from HIV-1 subtypes/CRFs previously reported in Iran including B (●; closed circles), D (□; open squares), A1 (■; closed squares), A2 (○; open circles), CRF01_AE (◆; closed diamonds) and CRF35_AD (▲; closed triangle) are included. GenBank accession numbers for the reference sequences, as well as, bootstrap values over 70% are shown. The scale bar represents nucleotidesubstitutions per site.

The baseline characteristics of the study cases, mean age 35.9 years of age with most cases being male (85%) and single (62.5%) are shown in Table 1. CD4+ T cell counts ranged from 50 to 682 cells/mm^3^ (median 428), and duration of HIV infection since the first detected seroconversion ranged from 2 to 9 years(median 4.5 years), and 37.5% of the cases were co-infected with HCV. Phylogenetic analysis of PR-RT sequences against the reference sequences of 9 subtypes and 72 CRFs revealed that all 40 samples (100%) were CRF35_AD (the summarized tree is shown in Fig 1). This was also supported by results obtained from analysis by the REGA Subtyping Tool (data not shown).

**Table 1:** Demographics and laboratory data of study samples.

Characteristics	Samples (n = 40)
**Demographics**	
Male	34 (85%)
Female	6 (15%)
Age, years, mean ± SD	35.9 ± 7.08
Marital status	
Single	25 (62.5%)
Married	11 (27.5%)
Divorced	4 (10%)
**Laboratory parameters**	
CD4+ cell count, cells/mm^3^, median (range)	428 (50–682)
Seroconversion time, year, median (range)	4.5 (2–9)
Hepatitis B virus antigen positive	0
Hepatitis C virus antibody positive	15 (37.5%)

SD, standard deviation

Among the detected DRAMs (Table 2), M41L and M184V that confer the clinical resistance to zidovudine (AZT), lamivudine (3TC), stavudine (d4T) and abacavir (ABC) were the most common to nucleoside reverse transcriptase inhibitors (NRTIs; 5%). K103N associated with resistance to efavirenz (EFV) and nevirapine (NVP) was the only mutation to non-nucleoside reverse transcriptase inhibitors (NNRTIs; 5%). Detection of these mutations is important in view of the fact that AZT, 3TC, EFV and NVP are among the most common first-line regimens in Iran. We found no SDRMs to protease inhibitors (PIs); however, based on the mutations listed in the International Antiviral Society-USA (IAS-USA) panel, 13minor mutations were detected in the PR region, of which M36I, H69K, and L89M/V/I were found in all 40 (100%) andK20R/T was detected in 37 (92.5%) samples. V11I, L33V and D60E that were already reported as minor PI DRAMs in CRF35_AD [18] were not detected in Sanandaj samples. Instead, T74S (2.5%) that is a common accessory mutation and V77I (2.5%) that confers moderate resistance to indinavir (IDV), nelfinavir (NFV) and saquinavir (SQV) were identified in our samples. Based on these data, the overall frequency of WHO-recommended SDRMs in the present population was 15%, which was classified to NRTIs (10%), NNRTIs (5%) and PIs (0%).

**Table 2:** Frequency of HIV-1 drug resistance-associated mutations among 40 sequenced samples.

SDRMs (based on WHO list)	*n* (Frequency)
**NRTI-resistant mutations**	
M41L	2 (5%)
D67N	1 (2.5%)
K70R	1 (2.5%)
V75M	1 (2.5%)
F116Y	1 (2.5%)
M184V	2 (5%)
L210W	1 (2.5%)
T215Y	1 (2.5%)
K219E	1 (2.5%)
At least one NRTI SDRM	4 (10%)
**NNRTI-resistant mutations**	
K103N	2 (5%)
At least one NNRTI SDRM	2 (5%)
**PI-resistant mutations**	None
Minor PI-resistant mutations (based on IAS-USA list)	
L10V/I/F	7 (17.5%)
V11I	1 (2.5%)
G16E	3 (7.5%)
K20R/T	37 (92.5%)
M36I	40 (100%)
I62V	4 (10%)
L63P	2 (5%)
I64L/M	2 (5%)
H69K	40 (100%)
T74S	1 (2.5%)
V77I	1 (2.5%)
L89M/V/I	40 (100%)
I93L	1 (2.5%)
Any DRAMs (excluding PI-resistant mutations)	6 (15%)

SDRMs, surveillance drug-resistant mutations; NRTIs, nucleoside reverse transcriptase inhibitors; NNRTIs, non-nucleoside reverse transcriptase inhibitors; PIs, protease inhibitors; DRAMs, drug resistance-associated mutations.

## Discussion

In this study we report the HIV-1 circulating subtype and the prevalence of TDR among 40 chronically infected cases in a Western city of Iran. Considering that IDUs comprise the majority of HIV-infected population in Iran [15–16]our samples were selected from a cohort of ART-naïve IDUs. All study cases were determined to be infected with the CRF35_AD variant. This finding is in support of previous studies reporting predominance of CRF35_AD among Iranian HIV-infected IDUs [18–19, 25]. CRF35_AD was originally identified in 2007 in Afghanistan[26] and since then has been reported as a circulating variant in Iran, Afghanistan and Pakistan [27–28].

Genotypic HIV-1 drug resistance can be interpreted using various available databases. Comparison of these algorithms for the interpretation of TDR has shown minor difference for resistance to PIs and NNRTIs, and more pronounced variations for resistance to NRTIs [29]. To address this concern, WHO recommends the use of the SDRMs standard list to make TDR results obtained from different regions, different times and different groups comparable [14]. Accordingly, the overall frequency of SDRMs we found in chronically infected subjects was 15% (NRTIs 10%, NNRTIs 5%, and PIs 0%). Compared to the previously reported TDR frequencies of 5.1% [19]and 4.2% [18] among Iranian newly infected subjects, the frequency of transmitted mutations in our samples appears relatively high although it still remains within the moderate prevalence (5–15%) previously calculated for the SDRMs in Iran [18].

In contrast to these earlier reports that identified a limited number of NRTI mutations (T215D, K219Q [18] and D67G, V75A [19]) with a low overall frequency (4.2% and 5.1%, respectively) in newly infected Iranian cases, we detected a variety of NRTI SDRMs (M41L, D67N, K70R, V75M, F116Y, M184V, L210W, T215Y, and K219E) with a higher overall frequency (10%) in chronically infected IDUs in the city of Sanandaj (Table 2). Furthermore, results from the two mentioned studies found no NNRTI SDRMs, while K103N was identified in 5% of our samples.

We did not find any WHO-recommended SDRMs within the protease region of our samples. However, based on the IAS-USA algorithm, several minor mutations were detected. Moreover, in agreement with previous reports[18–19] we identified high frequencies of M36I, H69K, L89M/V/I and K20R/T mutations that may be considered as polymorphic signatures within the protease region of CRF35_AD. The absence of major mutations to PIs was anticipated and previously reported in newly infected drug-naïve Iranian subjects, as well. The general consensus is that limited access to PIs in Iran has caused an uncommon transmission of PR mutations [18].

It is believed that to facilitate viral replication, initial mutations present in recent infections tend to revert to the wild-type overtime, and hence, in the absence of drug selection, a lower frequency of resistance-associated mutations are expected to be found in subjects with chronic infections compared to those recently infected[30–31]. On the contrary, there are studies reporting no substantial differences between subjects with acute and long-lasting infection [4, 32]. Herein, compared with two previous Iranian studies on recent infections we identified a higher frequency of SDRMs among individuals with chronic infection. Differences in cohort characteristics, as well as, time and region of sampling may have contributed to this discrepancy. Of note, previous studies worked on heterogeneous HIV infected populations from multiple geographical areas, while we focused on IDU samples from a single city. Furthermore, when disregarding the duration of infection, the fact that we selected our samples merely from a cohort of IDUs living in one city may explain the relatively high frequency of mutations we detected. It has been shown that high infection risk behaviors such as sharing syringes and needles, as well as, participating in unprotected sexual practice are frequent among Iranian IDUs [16]. These common risk behaviors in addition to living in one geographical area could result in circulation of mutant viruses in this population. Given this possibility, epidemiological linkage of the study participants may have increased the rate of TDR mutations detected in our study.

In conclusion, our survey shows that the transmission of RT inhibitor resistance occurs in a relatively high frequency in chronically infected HIV-1 positive IDUs in the city of Sanandaj raising the concern of treatment failure in this population. These results are further highlighted when compared to first-line ART in Iran, which since 2008 has been mainly based on the co-administration of NRTIs and NNRTIs. Our findings also emphasize the requirement for routine drug resistance testing to optimize first-line treatment in HIV-1 chronic infections. Our study was geographically limited to one city in the West of Iran. Therefore, in order to generalize these results, prospective drug resistance surveys should be considered in drug-naïve individuals with long-term established HIV-1 infection in other cities throughout Iran.
